# Loss of CD11b Accelerates Lupus Nephritis in Lyn-Deficient Mice Without Disrupting Glomerular Leukocyte Trafficking

**DOI:** 10.3389/fimmu.2022.875359

**Published:** 2022-05-12

**Authors:** Timothy A. Gottschalk, Pamela Hall, Evelyn Tsantikos, Elan L’Estrange-Stranieri, Michael J. Hickey, Margaret L. Hibbs

**Affiliations:** ^1^Leukocyte Signalling Laboratory, Department of Immunology and Pathology, Central Clinical School, Monash University, Melbourne, VIC, Australia; ^2^Centre for Inflammatory Diseases, Monash University Department of Medicine, Monash Medical Centre, Clayton, VIC, Australia

**Keywords:** systemic lupus erythematosus, glomerulonephritis, inflammation, myeloid cells, immune cell trafficking, Lyn tyrosine kinase, leukocyte integrin CD11b

## Abstract

Systemic lupus erythematosus (SLE) is a complex, heterogeneous autoimmune disease. A common manifestation, lupus nephritis, arises from immune complex deposition in the kidney microvasculature promoting leukocyte activation and infiltration, which triggers glomerular damage and renal dysfunction. CD11b is a leukocyte integrin mainly expressed on myeloid cells, and aside from its well-ascribed roles in leukocyte trafficking and phagocytosis, it can also suppress cytokine production and autoreactivity. Genome-wide association studies have identified loss-of-function polymorphisms in the CD11b-encoding gene *ITGAM* that are strongly associated with SLE and lupus nephritis; however, it is not known whether these polymorphisms act alone to induce disease or in concert with other risk alleles. Herein we show using *Itgam^-/-^* mice that loss of CD11b led to mild inflammatory traits, which were insufficient to trigger autoimmunity or glomerulonephritis. However, deficiency of CD11b in autoimmune-prone Lyn-deficient mice (*Lyn^-/-^Itgam^-/-^*) accelerated lupus-like disease, driving early-onset immune cell dysregulation, autoantibody production and glomerulonephritis, impacting survival. Migration of leukocytes to the kidney in *Lyn^-/-^* mice was unhindered by lack of CD11b. Indeed, kidney inflammatory macrophages were further enriched, neutrophil retention in glomerular capillaries was increased and kidney inflammatory cytokine responses were enhanced in *Lyn^-/-^Itgam^-/-^* mice. These findings indicate that *ITGAM* is a non-monogenic autoimmune susceptibility gene, with loss of functional CD11b exacerbating disease without impeding glomerular leukocyte trafficking when in conjunction with other pre-disposing genetic mutations. This highlights a primarily protective role for CD11b in restraining inflammation and autoimmune disease and provides a potential therapeutic avenue for lupus treatment.

## Introduction

Systemic lupus erythematosus (SLE, lupus) is a multi-organ, heterogeneous autoimmune disease mediated by autoreactive B cells that generate autoantibodies to nuclear components (anti-nuclear antibodies, ANA). These autoantibodies form immune complexes that deposit within the microvasculature of various tissues, eliciting an inflammatory response (1). The immune response is typified by broad systemic activation and dysregulation of the innate, adaptive and humoral systems, with inflammatory cytokines and chemokines driving localized responses, which invoke the recruitment and activation of pathogenic cells that damage the tissue (2). The kidney is a common target of aberrant inflammation in SLE patients, and the progression of the resultant nephritic injury to end-stage organ failure adds significantly to SLE-associated morbidity and mortality (3). As SLE is diverse both clinically (pathogenesis and presentation) and genetically (susceptibility risk loci), diagnosis and treatment can be challenging; therefore, further understanding of the complex pathogenic processes and genetic risk associations will aid advancements in patient care.

Genome-wide association studies have identified single nucleotide polymorphisms (SNPs) in the *ITGAM* locus, which encodes the protein CD11b, that are strongly associated with SLE (4–6). CD11b (α_M_) is a leukocyte integrin that heterodimerizes with the common β2 integrin subunit (CD18) to form the adhesion molecule, Mac-1 (α_M_β_2_), which is expressed mainly on leukocytes of the myeloid lineage (7). As an adhesion molecule, CD11b plays a key role in immune cell trafficking during inflammation, mediating leukocyte attachment to the vascular endothelium *via* interactions with numerous ligands, including ICAM-1 and VCAM-1 (8). In this capacity, CD11b shares a degree of functional redundancy with other members of the β2-pairing integrin family: CD11a (α_L_β_2_, LFA-1), CD11c (α_X_β_2_, p150/95) and CD11d (α_D_β_2_) (9–11). However, CD11b has various specialized non-trafficking functions that promote inflammatory responses, prominently through facilitating iC3b complement-mediated phagocytosis, supporting FcγR-mediated processes including immune complex-mediated phagocytosis, antibody-dependent cellular cytotoxicity and NETosis, and activating neutrophil cytotoxic processes (10, 12, 13). Conversely, CD11b is also immunoregulatory, and can suppress autoreactivity through phagocytosis of cellular debris and immune complexes and suppression of autoimmune B cells (14) and myeloid inflammatory cytokine responses (15–17). As CD11b has several dichotomous functions, it has the potential to act in various capacities to both regulate and propagate immunopathology in lupus. Assessment of the common lupus-susceptibility variants in CD11b show diminished phagocytic capacity (18, 19), suggesting loss of function is associated with disease. However, the impact of CD11b-deficiency on lupus nephritis has not been extensively explored in spontaneous, chronic *in vivo* models, and it is unclear whether loss-of-function CD11b mutations are a monogenic risk for lupus.

Mice lacking the immunoregulatory Src family tyrosine kinase, Lyn (*Lyn*^-/-^), spontaneously develop immune cell defects, antinuclear antibodies, systemic inflammation, and immune-complex mediated glomerulonephritis, making it a robust pre-clinical model of lupus (20, 21). *Lyn^-/-^* mice also show dysregulation of CD11b, suggesting that this may contribute to disease (22). Therefore, to examine the influence of CD11b on lupus pathology, we generated Lyn^-/-^ mice lacking CD11b (*Lyn^-/-^Itgam^-/-^*). We show that loss of CD11b alone is insufficient to drive autoimmune pathology, but on a Lyn-null genetic background, CD11b-deficiency accelerates systemic inflammation and local inflammatory responses in the kidney, exacerbating autoimmune kidney pathology without interfering with glomerular leukocyte trafficking. This study shows that CD11b-deficiency is associated with lupus in a non-monogenetic manner and CD11b can be dispensable for leukocyte trafficking in chronic renal disease.

## Materials and Methods

### Animals

C57BL/6 background *Lyn^-/-^* mice (L^-/-^) (20) and *Itgam*^tm1Myd^ mice (*Itgam*^-/-^, M^-/-^) (23) were crossed to generate *Lyn^-/-^Itgam^-/-^* double deficient mice (LM^-/-^) and confirmed by genotyping. Mice were bred under specific pathogen-free conditions at the Monash University Animal Research Platform (Clayton, Australia) and housed at the Monash University Intensive Care Unit (Melbourne, Australia) for long-term studies. C57BL/6 control mice were obtained from Monash Animal Services (Clayton, Australia). Research was performed in accordance with the Australian Code for the Care and Use of Animals for Scientific Purposes and in agreement with NHMRC animal welfare guidelines. Approval for animal experimentation was obtained from the Alfred Research Alliance (E/1377/2013/M, E/1688/2016/M) and Monash Medical Centre B (2018/19) animal ethics committees.

### Flow Cytometry

Single cell suspensions of spleen were prepared by extrusion. Saline-perfused kidneys were digested in RPMI + 0.1 mg/ml Liberase TL (Roche, 5401020001) + 0.1 mg/ml DNAse I at 37°C then mechanically dissociated to form a single cell suspension. Cells were treated with ACK red blood cell lysis buffer, filtered, counted and Fc receptors were blocked with anti-FcγRII/III F(ab’)_2_ (2.4G2, in-house). Cells were then stained with fluorophore-conjugated mAbs and acquired on an LSRFortessa (BD Biosciences) with FACSDiva™ software (BD Biosciences) and analyzed using FlowJo Software (BD Bioscience). Dead cells were excluded based on uptake of propidium iodide or FluoroGold. Splenic cell populations were defined as: erythroblasts: Ter119^+^CD71^±^; monocytes CD115^+^Ly6G^-^; neutrophils: Ly6G^+^CD115^-^; CD4^+^ T cells: CD4^+^CD8α^-^; CD8^+^ T cells: CD8α^+^CD4^-^. B cells: B220^+^CD138^low^; plasma cells: B220^low-mid^CD138^high^. Monocytes were further classified as conventional/inflammatory: Ly6C^+^CD62L^+^; non-conventional/patrolling: Ly6C^-^CD62L^-^. CD4^+^ and CD8^+^ T cells were further classified as effector cells: CD44^+^CD62L^-^. B cells were further subdivided as follicular: CD23^+^CD21^low^; marginal zone: CD23^-^CD21^+^; or transitional: CD23^-^CD21^-^. Kidney lymphocytes were pre-gated CD45^+^CD11b^-^CD11c^-^ and defined as: γδ T cells: CD3ϵ^+^γδTCR^+^; CD4^+^ T cells: γδTCR^-^CD3ϵ^+^CD4^+^; CD8^+^ T cells: γδTCR^-^CD4^-^CD3ϵ^+^CD8α^+^; B cells: γδTCR^-^CD4^-^CD8α^-^CD19^+^. Kidney granulocytes were pre-gated CD45^+^F4/80^-^CX3CR1^-^ and defined as: eosinophils: SiglecF^+^Ly6G^-^; neutrophils: Ly6G^+^SiglecF^-^. Kidney mononuclear phagocytes were pre-gated CD45^+^F4/80^+^CX3CR1^+^ and defined as: resident macrophage-like phagocytes: CD11c^+^Ly6C^-^; inflammatory macrophages: CD11c^+^Ly6C^+^. Kidney endothelium was defined as CD45^-^CD326^-^CD31^+^. Absolute cell numbers from spleen were determined from total cell counts and cell proportions identified by flow cytometry; cell numbers from kidney were determined by flow cytometry gated event count outputs and proportion of whole tissue acquired. Expression of activation and phenotypic markers (CD11b, CD62L, FcγRII/III, IgM) were determined by normalizing the geometric mean fluorescence intensity (gMFI) by dividing the value of each sample by the mean value of the C57BL/6 group within each experiment, allowing pooling of data from multiple experiments. Negative expression controls for activation markers were derived from gating on the population of C57BL/6 splenocytes that do not express the marker.

### Kidney Histopathology

Kidney histopathology was conducted as previously described (22). Briefly, formalin-fixed, paraffin-embedded sections of 3 µm thickness were stained with hematoxylin and eosin or Periodic Acid-Schiff (PAS) for histopathological analysis. PAS-stained slides were imaged by Aperio Scanscope AT Turbo (Leica Biosystems). Mean glomerular cross-sectional area was quantified by polygon tracing using ImageJ software (NIH, 1.52d). Mean disease score criteria were assessed blinded as follows; 0 – Normal glomerular cellularity and morphology; 1 – Mild cellular expansion and/or early-stage disrupted glomerular morphology which includes lobularity and/or Bowman’s space enlargement; 2 – Advanced cellular expansion with distinct disruption to glomerular morphology, pyknosis and/or karyorrhexis; 3 – Severe cellular expansion/consolidation with advanced lobularity or fragmentation and/or enlargement of the Bowman’s space with cellular infiltration (with or without crescent formation) and/or peri-glomerular cellular expansion and/or evidence of segmental necrosis; 4 – End-stage glomerular destruction, progressive or complete loss of cellularity and/or distinct glomerular morphology. At least 30 glomeruli were assessed for each kidney.

### Survival Study

Ageing cohorts of sex-matched *Lyn^-/-^* (n=18) and *Lyn^-/-^Itgam^-/-^* (n=19) mice received daily condition checks and were euthanized when their condition had deteriorated to predefined ethical checkpoints set by researchers in consultation with veterinary staff. Lifespan in days were recorded at these time-points and graphed as a Kaplan-Meier survival curve.

### Autoantibody Quantification by ELISA

Detection of autoantibodies was conducted as previously described (22). Briefly, 96-well plates (Maxisorp, Nunc) were coated with calf thymus DNA (Sigma-Aldrich). After incubation of serum samples and reference serum, goat anti-mouse IgG(H+L)-HRP (Southern Biotech) detection antibody followed by TMB chromogenic substrates A and B (BD Biosciences) elicited a colorimetric change which was recorded by a MultiSkan GO microplate spectrophotometer (Thermo Fisher Scientific). Relative titers were determined by plotting sample optical density against the reference serum-derived standard curve.

### Quantitative Real-Time PCR

RNA was extracted from frozen kidney tissue by trizol and chloroform separation with genomic DNA removal by DNA-free DNA removal kit (Thermo Fisher). cDNA was generated by FireScript RT kit (Solis Biodyne) as per manufacturer’s instructions. RT-PCR was performed with validated primers for *Gapdh* (housekeeping control) (24), *Il1b* (25), *Tnfa* (Fwd 5’ CCCTCACACTCAGATCATCTTCT 3’, Rev 5’ GCTACGACGTGGGCTACAG 3’), *Ccl2* (26), *Cxcl1* (27) and PowerUp SYBR Green Master Mix (Applied Biosystems) for 40 cycles under single-plex conditions (QuantStudio 6, Life Technologies). cDNA-specific amplification was ensured by inclusion of reverse transcriptase-negative and template-negative controls. Samples were run in triplicate and cycle threshold values were determined by automatic threshold analysis (QuantStudio Software), with the 2^-ΔΔCt^ method used to calculated relative gene expression from the average threshold of each sample, relative to the average of the C57BL/6 group.

### Assessment of Kidney Leukocyte Infiltration by Immunohistochemistry

Formalin-fixed paraffin-embedded kidney sections (3 µm) underwent antigen-retrieval in DAKO antigen-retrieval buffer (Agilent). Sections were blocked with PBS/1% BSA, followed by incubation with rat anti-mouse CD45-biotin, then anti-rat-HRP secondary antibody (both in PBS/0.2% BSA). Chromogenic substrate (DAKO diaminobenzidine chromogen solution; Agilent) was added to sections followed by counter-staining with hematoxylin and coverslip mounting with DPX (Sigma-Aldrich). Sections were imaged using an Olympus BX-51 light microscope at 20x objective magnification (Olympus Australia), capturing at least six images of each kidney cortex. Images were analyzed using ImageJ software by isolating the red channel and setting the color threshold to 120 to visualize punctate staining. CD45+ staining was quantified using the polygon tool to trace each glomerulus and measuring the area fraction. A minimum of 30 glomeruli were analyzed per kidney, with the mean taken to represent each sample.

### Assessment of Glomerular IgG Deposition by Immunofluorescence

Saline-perfused kidneys were fixed in PLP fixative (PBS + 1.37%w/v L-lysine + 2%w/v paraformaldehyde + 0.22%w/v sodium periodate), infused with 30%w/v sucrose solution then embedded and snap frozen in OCT. 7 μm kidney sections were cut and mounted on SuperFrost Plus slides, rehydrated in PBS, blocked (4% BSA + 0.05% sodium azide in PBS) then stained with 20 μg/mL goat anti-mouse IgG-AF488 (*In vivo*gen, A11029). Stained sections were mounted with antifade (4%w/v n-propyl gallate in 90% glycerol + 10% PBS), cover-slipped and imaged using the Nikon Eclipse TE2000-U Inverted Fluorescence Microscope with DS-Ri2 camera and 470/40 filter. IgG deposition was quantified in a blinded fashion with ImageJ FIJI, using the polygon tool to trace the glomerular tissue and measuring the mean gray value. A minimum of 30 glomeruli were analyzed per kidney, with the mean taken to represent each sample.

### Kidney Intravital Multiphoton Microscopy

Glomerular trafficking of neutrophils and Ly6C^+^ monocytes was assessed by intravital multiphoton microscopy using a modification of a previously published technique (28–30). In brief, mice were anesthetized with ketamine hydrochloride (150 mg kg^-1^)/xylazine hydrochloride (10 mg kg^-1^), a catheter was inserted into the jugular vein, and the left kidney exteriorized *via* a dorsal incision. The intact kidney was immobilized in a heated well incorporated in a custom-built stage, bathed in normal saline and cover-slipped. The renal microvasculature was visualised using an FVMPE-RS multiphoton microscope (Olympus Australia, Notting Hill, Vic.), equipped with a 25X 1.05 NA water-immersion objective and an InSight X3 laser (Spectra-Physics, Milpitas, CA) tuned to 800 nm. Two recordings of superficial glomeruli were made for 30 min in each mouse, recording images every 30 s, collecting z-stack images of ~100 µm depth at a step-size of 3 μm. Emitted fluorescence was collected in non-descanned detectors with 410-455 nm, 495-540 nm and 575-645 nm emission filters. The glomerular microvasculature was labelled with AlexaFluor (AF) 594-anti-CD31 (clone MEC 13.3, BioLegend, San Diego, CA; 2 μg), adherent neutrophils with AF488-anti-Ly6G (1A8, eBioscience, Scoresby, Vic.; 2 μg) and Ly6C^+^ monocytes with AF405-anti-Ly6C (HK1.4, Novus; 3 μg), all administered i.v. immediately prior to imaging. Glomeruli were located on the basis of anti-CD31 staining, and the adhesion (cells/glomerulus/30 min) and duration of intraglomerular retention (dwell; min) determined for neutrophils and Ly6C^+^ monocytes using *Imaris* (Bitplane, Zurich).

### Statistical Analyses

Statistical significance was determined using Mann-Whitney non-parametric U test (for two groups) or Kruskal-Wallis H test followed by Dunn’s multiple comparisons test (for three groups). The *Itgam^-/-^* group, acting as a reference control, was excluded from multiple comparison tests. To confirm an intermediate phenotype, specific comparisons between the *Lyn^-/-^* and C57BL/6 or *Lyn^-/-^Itgam^-/-^* groups were also conducted using Mann-Whitney non-parametric U test. Horizontal bars represent median ± IQR where the data are displayed on a linear scale, or geometric mean ± geometric SD where logarithmic data are displayed. Significance by Mann-Whitney test (represented by #) and Dunn’s multiple comparisons test (represented by *) is denoted by p > 0.05 (not significant) ns or not stated, p < 0.05 # or *, p < 0.01 ## or **, p < 0.001 ### or ***, p < 0.0001 #### or ****. Significance of the survival study was determined by log-rank (Mantel-Cox) and Gehan-Breslow-Wilcoxon tests where p < 0.05 is significant. Statistical analysis was conducted by GraphPad Prism software (version 9.0.1).

## Results

### CD11b-Deficiency Alone Is Insufficient to Drive Autoimmune Disease

**Figure 1:**
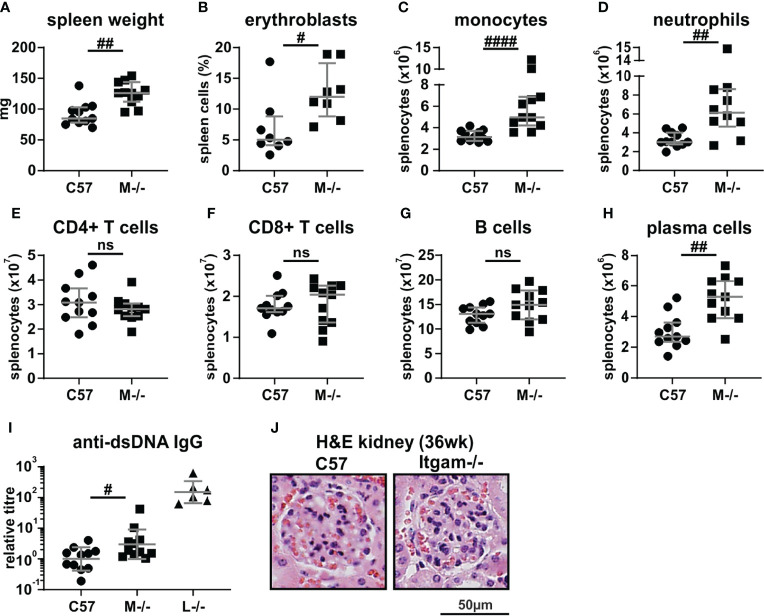
CD11b-deficiency elicits immune compartment changes but not autoimmune pathology. 36-week-old C57BL/6 and *Itgam^-/-^* mice were assessed for: **(A)** spleen weights; flow cytometric quantitation of splenic populations of **(B)** erythroblasts, **(C)** monocytes, **(D)** neutrophils, **(E)** CD4+ and **(F)** CD8+ T cells, **(G)** B cells and **(H)** plasma cells; **(I)** quantitation of systemic titers of anti-dsDNA IgG autoantibodies (with age-matched *Lyn^-/-^* (n = 6) positive controls); and **(J)** glomerular histopathology. For **(A–I)**, n = 8-11 mice per group, pooled from four experiments. The images in **(J)** are representative of n = 8-11 mice per group. Not significant denoted as ns, ^#^p < 0.05, ^##^p < 0.01, ^####^p < 0.0001 by Mann-Whitney U test.

Given the observations of increased susceptibility to SLE in individuals bearing *ITGAM* SNPs, we first determined whether deficiency of CD11b in mice was sufficient to drive changes in the immune system consistent with development of autoimmunity. *Itgam^-/-^* mice were assessed at 36 weeks of age, a time point where autoimmune disease development is severe in lupus-prone *Lyn^-/-^* mice (22, 31). Aged *Itgam^-/-^* mice exhibited splenomegaly (**Figure 1A**), due to expansion of splenic erythroblasts, monocytes, and neutrophils (**Figures 1B–D**). While CD4^+^ T cells, CD8^+^ T cells and B cells were unchanged (**Figures 1E–G**), plasma cells were significantly expanded in *Itgam^-/-^* mice (**Figure 1H**), although this correlated with only slight increases in autoantibody titers (**Figure 1I**). Histopathological assessment of kidneys revealed healthy glomeruli with no indication of cellular or morphological changes or enlargement of glomeruli in 36-wk old *Itgam*^-/-^ mice (**Figure 1J**). These findings indicate that while some key effector immune cell populations are expanded in 36-week-old *Itgam*^-/-^ mice, no overt autoimmunity or immunopathology is evident at this time.

### Loss of CD11b Accelerates Autoimmune Disease and Inflammatory Traits in *Lyn*^-/-^ Mice

**Figure 2:**
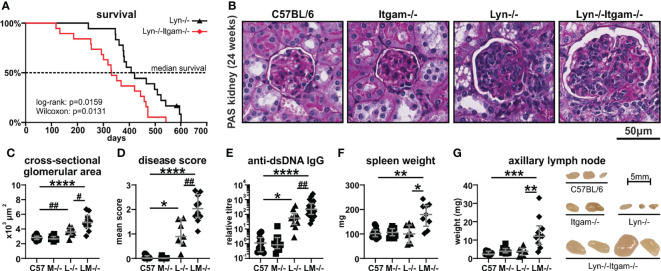
*Lyn^-/-^Itgam^-/-^* mice exhibit enhanced systemic autoimmune disease. **(A)** Survival of *Lyn^-/-^* and *Lyn^-/-^Itgam^-/-^* mice by Kaplan-Meier survival analyses. The indicated 24-week-old mice were evaluated for: **(B)** glomerular histopathology by PAS staining with quantitation of **(C)** glomerular cross-sectional area and **(D)** mean disease score; **(E)** anti-dsDNA IgG autoantibodies; **(F)** spleen weight and **(G)** axillary lymph node weight and size. For **(A)**, n = 18-19 per group, significance determined by log-rank and Wilcoxon tests. Data in **(C, D, F ,G)** are of n = 7-12 per group pooled from three experiments and data in **(E)** are of n = 10-21 per group. Images in **(B, G)** are representative of three experiments. *p < 0.05, **p < 0.01, ***p < 0.001, ****p < 0.0001 by Dunn’s multiple comparisons test and ^#^p < 0.05, ^##^p < 0.01 by Mann-Whitney U test.

Lupus-prone *Lyn^-/-^* mice exhibit intrinsic CD11b dysregulation, with neutrophils showing upregulated CD11b from a pre-disease age (12 weeks) and throughout early- (24 weeks) and late-stage (36 weeks) disease (**Supplementary Figure 1A**) (22), suggesting that CD11b may contribute to early and ongoing disease processes. Therefore, to establish whether CD11b influences the development of inflammation and autoimmune disease on an autoimmune-susceptible genetic background, *Lyn^-/-^Itgam^-/-^* mice (LM-/-) were generated and assessed. Initial survival studies revealed that *Lyn^-/-^Itgam^-/-^* mice had significantly poorer survival (median 332 days) compared to *Lyn^-/-^* mice (median 414.5 days) (**Figure 2A**). Histopathological analysis of kidneys at 36 weeks of age, where disease is typically maximal in *Lyn^-/-^* mice (22), showed similarly severe glomerular damage in both *Lyn^-/-^* and *Lyn^-/-^Itgam^-/-^* mice (**Supplementary Figure 1B**). However, assessment of renal pathology at 24 weeks of age, which captures early and progressing disease stages, showed that *Lyn^-/-^Itgam^-/-^* mice already exhibited significantly advanced glomerular enlargement (median area 4885 µm, IQR 4198-5849), lobularity and cellular expansion and periglomerular dysplasia (median disease score 2.029, IQR 1.676-2.612) well beyond the mild disease seen in *Lyn^-/-^* mice (median glomerular area 3590 µm, IQR 3235-4208; median disease score 0.894, IQR 0.600-1.611) (**Figures 2B–D**). Systemic indices of autoimmunity and inflammation were also assessed, and at 24 weeks of age, autoantibody titers were significantly elevated in *Lyn^-/-^Itgam^-/-^* mice compared to *Lyn^-/-^* mice (**Figure 2E**), and both splenomegaly (**Figure 2F**) and lymphadenopathy (**Figure 2G**) were pronounced in *Lyn^-/-^Itgam^-/-^* mice only. Collectively, these findings indicate that CD11b-deficiency accelerates systemic autoimmunity and renal disease progression in *Lyn^-/-^* mice.

### CD11b-Deficiency Further Enhances Peripheral Immune Cell Defects in *Lyn^-/-^* Mice

**Figure 3:**
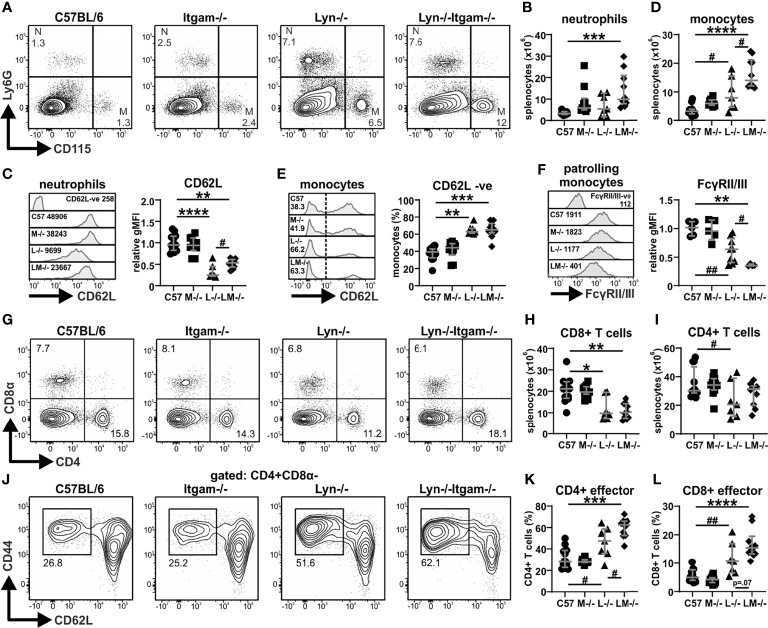
CD11b-deficiency augments splenic immune cell defects in *Lyn^-/-^* mice. Spleens from the indicated groups of 24-week-old mice were assessed by flow cytometry for: **(A)** proportions of neutrophils (Ly6G^+^CD115^-^) and monocytes (CD115^+^Ly6G^-^); **(B)** numbers of neutrophils with **(C)** relative expression of CD62L on neutrophils; **(D)** numbers of monocytes with **(E)** proportions of CD62L- non-conventional/patrolling monocytes and **(F)** relative expression of Fcγ receptors II and III on patrolling monocytes; **(G)** proportions of CD8+ (CD8α^+^CD4^-^) and CD4+ (CD4^+^CD8α^-^) cells with numbers of **(H)** CD8+ T cells and **(I)** CD4+ T cells; **(J)** proportions of effector (CD44^+^CD62L^-^) CD4+ T cells with numbers of **(K)** CD4+ effector T cells and **(L)** CD8+ effector T cells. For data in **(B–F, H, I, K, L)**, n = 7-12 per group, pooled from three experiments. Flow plots and histograms in **(A, C, E–G, J)** are representative of three experiments. The negative expression control histograms (CD62L-ve in **C**, FcγRII/III-ve in **F)** were derived by gating on C57BL/6 splenocytes that do not express the respective markers. *p < 0.05, **p < 0.01, ***p < 0.001, ****p < 0.0001 by Dunn’s multiple comparisons test and p value stated if approaching significance (p < 0.1), ^#^p < 0.05, ^##^p < 0.01 by Mann-Whitney U test.

To assess the cellular impacts of CD11b-deficiency, flow cytometry was performed on splenocytes. Lyn^-/-^ mice with late-stage disease typically present with expanded splenic myeloid cell populations (22, 32), however at 24 weeks, neutrophils (Ly6G+CD115-) were only expanded in *Lyn^-/-^Itgam^-/-^* but not *Lyn^-/-^* mice (**Figures 3A, B**), with both groups exhibiting downregulation of CD62L (**Figure 3C**). CD11b-deficiency further exacerbated expansion of splenic monocytes (CD115+Ly6G-) in *Lyn^-/-^* mice (**Figures 3A, D**), with both *Lyn^-/-^* and *Lyn^-/-^Itgam^-/-^* mice exhibiting a similar skewing toward CD62L-negative patrolling monocytes (**Figure 3E**). Interestingly, expression of FcγRII/III on these patrolling monocytes, which has implications for clearance of immunogenic cellular-debris and immune complexes, was progressively downregulated in *Lyn^-/-^* mice, yet more markedly lost on *Lyn^-/-^Itgam^-/-^* cells (**Figure 3F**). Together, this shows that CD11b-deficiency in *Lyn^-/-^* mice further promotes myeloid cell dysregulation.

Assessment of the T cell compartment revealed that *Lyn*^-/-^ mice displayed a typical age-associated T cell deficit, however, in 24-week-old *Lyn^-/-^Itgam^-/-^* mice, this was observed only in the CD8+ compartment and not the CD4+ compartment (**Figures 3G-I**). Skewing of the CD4+ T cell population towards effector cells (CD44^hi^CD62L^-^), which is typical in *Lyn^-/-^* mice with disease progression (22), was further enhanced in *Lyn^-/-^Itgam^-/-^* mice (**Figures 3J, K**), with a similar trend for CD8+ effector T cells (**Figure 3L**), indicating that CD11b-deficiency can further promote T cell activation phenotypes in *Lyn^-/-^* mice.

Assessment of the B cell compartment revealed that *Lyn^-/-^Itgam^-/-^* mice exhibited splenic B lymphopenia (**Supplementary Figures 2A, B**) and maturational defects (**Supplementary Figure 2C**) typical of *Lyn^-/-^* mice. Similarly, plasmacytosis was pronounced in *Lyn^-/-^Itgam^-/-^* mice (**Supplementary Figures 2A, D**), with a typical *Lyn^-/-^* phenotype; hyper-IgM and augmented FcγRII expression (**Supplementary Figures 2E, F**). Together, these data indicate that loss of CD11b has no overt influence on the intrinsic B cell and plasma cell defects in *Lyn^-/-^* mice.

### Inflammatory Responses in the Kidney Are Further Enhanced by CD11b-Deficiency in *Lyn^-/-^* Mice

**Figure 4:**
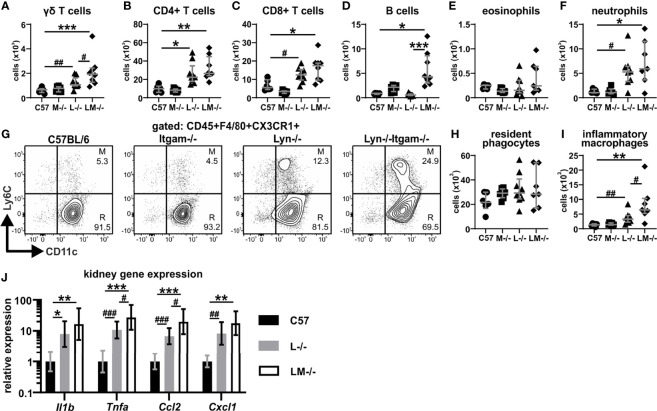
Kidney inflammatory responses are enhanced by CD11b-deficiency in *Lyn^-/-^* mice. Perfused and digested kidneys of 24-week-old mice were assessed by flow cytometry for numbers of: **(A)** γδ T cells, **(B)** CD4+ and **(C)** CD8+ T cells, **(D)** B cells, **(E)** eosinophils and **(F)** neutrophils; **(G)** populations of phagocytes gating on CD45+F4/80+CXCR3+ cells to delineate numbers of **(H)** resident macrophage-like phagocytes (CD11c+Ly6C-, R) and **(I)** inflammatory macrophages (CD11c+Ly6C+, M); **(J)** qRT-PCR gene expression of 24-week-old kidneys for *Il1b*, *Tnfa*, *Ccl2* and *Cxcl1*. For data in **(A–F, H, I)**, n = 5-9 per group, pooled from two experiments. Flow plots in **(G)** are representative of two experiments. Data in **(J)** are of n = 5-8 per group. *p < 0.05, **p < 0.01, ***p < 0.001 by Dunn’s multiple comparisons test and ^#^p < 0.05, ^##^p < 0.01, ^###^p < 0.001 by Mann-Whitney U test.

Given that CD11b-deficiency accelerates autoimmunity and lupus nephritis in *Lyn^-/-^* mice (**Figure 2**), we next examined the inflammatory responses in the kidney of 24-week-old mice. Flow cytometry was used to identify and quantify kidney immune cell populations (**Supplementary Figure 3A**). Total CD45+ leukocyte numbers in *Lyn^-/-^* kidneys were elevated above those in healthy C57BL/6 mice with a trend towards further expansion in *Lyn^-/-^Itgam^-/-^* mice (**Supplementary Figure 3A**). In comparison to healthy controls, *Lyn^-/-^* mice exhibited an expansion of γδ, CD4+ and CD8+ T cell subsets (**Figures 4A–C**), with almost no detection of intrarenal B cells (**Figure 4D**). A similar expansion in kidney lymphocytes was observed in *Lyn^-/-^Itgam^-/-^* mice, with the notable further enrichment of γδ T cells, and marked B cell infiltration (**Figures 4A–D**). Eosinophils were barely detected in kidneys across all groups (**Figure 4E**) while neutrophils were equally expanded in both *Lyn^-/-^* and *Lyn^-/-^Itgam^-/-^* kidneys (**Figure 4F**). Numbers of kidney-resident macrophage-like phagocytes (gated as CD45+F4/80+CX3CR1+CD11c+Ly6C-) were similar across all groups (**Figures 4G, H**), while infiltrating inflammatory macrophages (gated as CD45+F4/80+CX3CR1+CD11c+Ly6C+), were enriched in *Lyn^-/-^* kidneys but significantly further expanded in *Lyn^-/-^Itgam^-/-^* kidneys (**Figures 4G, I**). Together, these data indicate that the absence of CD11b enhances immune cell infiltration in *Lyn^-/-^* kidneys.

To examine this further, local inflammatory cytokine production in the kidney was measured by qRT-PCR. The inflammatory profile of the kidney in *Lyn^-/-^* mice was typified by increases in the proinflammatory cytokines *Il1* and *Tnfa* and the monocyte-recruiting *Ccl2*, and neutrophil-recruiting *Cxcl1* chemokines (**Figure 4J**). This was also observed in *Lyn^-/-^Itgam^-/-^* kidneys, yet with further significant increases in the expression of *Tnfa* and *Ccl2* above *Lyn^-/-^* mice (**Figure 4J**). These observations provide evidence that loss of CD11b leads to enhanced inflammatory responses in the kidneys of *Lyn^-/-^* mice.

### Leukocyte Trafficking to the Kidney Is not Perturbed by CD11b-Deficiency in *Lyn^-/-^* Mice

**Figure 5:**
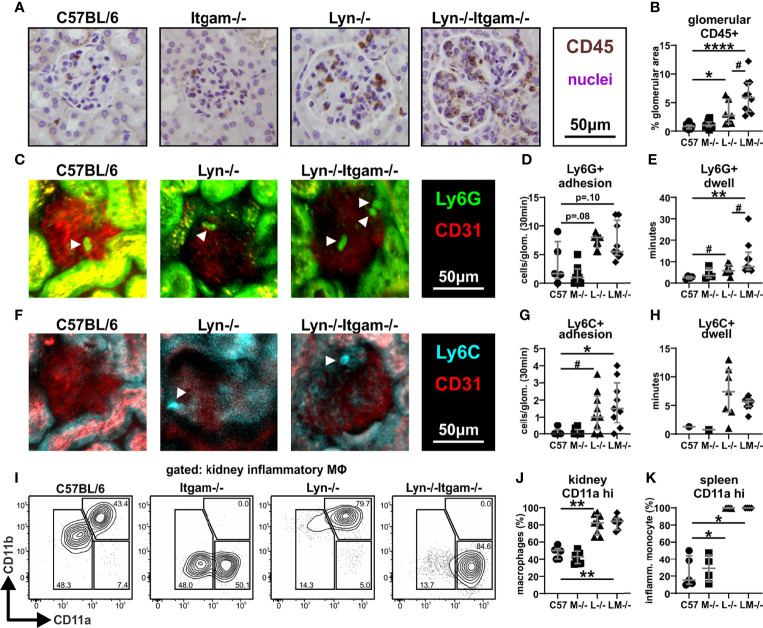
Leukocyte trafficking to the kidney is not perturbed by deficiency of CD11b in *Lyn^-/-^* mice. **(A)** Kidneys from 24-week-old mice were assessed by immunohistochemistry for the presence of CD45+ cells with **(B)** quantitation of CD45+ staining within the glomeruli. Two-photon live fluorescence imaging of the glomerular vasculature of 24-week-old mice depicting **(C)** Ly6G+ labelled neutrophils with quantification of **(D)** neutrophil adhesion (number of cells adhering to the vascular endothelium within a 30-minute window per glomerulus) and **(E)** Ly6G+ neutrophil dwell time (average length of time each detected cell remains adhered to the vascular endothelium). Two-photon live fluorescence imaging of the glomerular vasculature depicting **(F)** Ly6C+ labelled migratory monocytes with quantification of **(G)** migratory monocyte adhesion and **(H)** monocyte dwell time. **(I)** Flow cytometric assessment of the expression of integrin CD11a (LFA-1) on kidney Ly6C**+** inflammatory macrophages with **(J)** quantification and **(K)** quantification of CD11a expression on splenic Ly6C+ migratory monocytes. Images in **(A, C, F)** are representative of 3-5 experiments, white arrowheads indicate intravascular Ly6G+ or Ly6C+ cells. Data in **(B)** represent n = 7-12 per group and data in **(D, E, G, H)** are of n = 5-9 per group. Flow plots in **(I)** are representative of two experiments, data in **(J)** are of n = 5-9 per group and data in **(K)** are of n = 4-5 per group, both pooled from two experiments. *p < 0.05, **p < 0.01, ****p < 0.0001 by Dunn’s multiple comparisons test and p value stated if approaching significance (p < 0.1), ^#^p < 0.05, by Mann-Whitney U test.

Given that *Lyn^-/-^Itgam^-/-^* mice exhibited increased immune cells in the kidney which counters our previous observations that CD11b is required for the recruitment of cells to the kidney in acute inflammatory settings (28), we investigated renal leukocyte trafficking in *Lyn^-/-^Itgam^-/-^* kidneys at 24 weeks of age. Immunohistochemistry staining of CD45+ leukocytes revealed that infiltration in the kidney was largely focused to the glomeruli, or peri-glomerular areas with notable tissue dysplasia, which was most frequently observed in *Lyn^-/-^Itgam^-/-^* mice (**Figure 5A**). When quantified, *Lyn^-/-^* mice exhibited a small increase in glomerular leukocyte infiltration at this time-point, however this was elevated significantly further in *Lyn^-/-^Itgam^-/-^* mice (**Figure 5B**). Glomerular immune-complex deposition assessment showed similar elevation in both *Lyn^-/-^* and *Lyn^-/-^Itgam^-/-^* kidneys (**Supplementary Figure 4**), indicating that this is not driving the increased kidney leukocyte infiltration observed in *Lyn^-/-^Itgam^-/-^* mice.

Intravital multiphoton microscopy was employed to visualize leukocyte recruitment and interaction with the vasculature in the glomeruli of 24-week-old mice. The number of adherent Ly6G+ neutrophils in glomerular capillaries did not significantly differ across all groups (**Figures 5C, D**, **Supplementary Video 1**). Neutrophil retention (dwell time), which serves as a sensitive readout of the degree of glomerular inflammation (28–30), was mildly elevated in *Lyn^-/-^* kidneys compared to C57BL/6 kidneys, yet significantly further increased in *Lyn^-/-^Itgam^-/-^* mice (**Figure 5E**, **Supplementary Video 1**). Intraglomerular adhesion and retention of Ly6C+ inflammatory monocytes, which are less inclined to patrol the vasculature but can give rise to inflammatory kidney macrophages (33), was rare in C57BL/6 and *Itgam^-/-^* mice, yet was similarly enhanced in both *Lyn^-/-^* and *Lyn^-/-^Itgam^-/-^* kidneys (**Figures 5F–H**, **Supplementary Video 2**). Together these findings demonstrate that in Lyn-deficient mice, CD11b is not required for intraglomerular adhesion of neutrophils and monocytes, and that the combined absence of Lyn and CD11b leads to increased neutrophil retention in glomeruli.

To further explore the mechanisms of inflammatory leukocyte trafficking, the induction of adhesion and trafficking molecules in the kidney was assessed by flow cytometry. On CD31+ renal microvascular endothelial cells, expression of VCAM-1 was ubiquitous in all groups, however ICAM-1 was significantly upregulated in *Lyn^-/-^* and *Lyn^-/-^Itgam^-/-^* kidneys, albeit similarly in both groups (**Supplementary Figure 3B**), indicating comparable activation of the renal endothelium in *Lyn^-/-^* and *Lyn^-/-^Itgam^-/-^* mice. Assessment of the β2-pairing integrin family on renal immune cells revealed high surface expression of CD11a and an absence of CD11c on kidney neutrophils, regardless of CD11b expression (**Supplementary Figure 3C**). Ly6C+ inflammatory kidney macrophages (which are gated CD11c+), exhibited a marked upregulation of CD11a, dependent on loss of *Lyn* but not *Itgam* (i.e., on *Lyn^-/-^* and *Lyn^-/-^Itgam^-/-^* cells) (**Figures 5I, J**), and this phenotype was also observed in the peripheral precursor Ly6C+ monocytes (**Figure 5K**). Altogether, these findings demonstrate that during chronic inflammation, CD11b is dispensable for leukocyte recruitment to the diseased kidney and suggest that CD11a is the dominant trafficking integrin in *Lyn^-/-^* mice.

## Discussion

In this study, we examined the contribution of the leukocyte integrin CD11b in lupus-like autoimmune disease in mice, both as a stand-alone genetic deficiency, and in combination with Lyn-deficiency, a well-described lupus-susceptible model. In humans, two common SLE-associated CD11b variants from non-synonymous SNPs in the *ITGAM* locus have been described: R77H (rs1143679) confers an arginine to histidine substitution in the ligand-binding extracellular β propeller region, and P1146S (rs1143683) substitutes proline for serine in the cytoplasmic tail (18). Functional assessment of these variants showed impaired phagocytosis in neutrophils and monocytes without influencing the surface expression or activation of CD11b (18, 19), indicating loss-of-function, suggesting that CD11b function is largely protective against development of autoimmune pathology. As typically observed in spontaneous autoimmune-prone models, we demonstrate that deficiency of CD11b alone progressively drives some traits indicative of modest immune dysregulation and inflammation with age, yet *Itgam^-/-^* mice exhibit only very mild autoreactivity, which was insufficient to manifest typical autoimmune pathology. While this suggests that *Itgam*-deficiency is not a dominant monogenetic risk for lupus nephritis, it remains possible that *Itgam^-/-^* mice may progressively develop glomerulonephritis much later in life or with additional inflammatory triggers. Nonetheless, when coupled with Lyn-deficiency, CD11b-deficiency accelerated inflammation and autoimmune pathology, indicating that loss of functional CD11b is deleterious in inflammatory autoimmune disease settings. SLE is a notoriously heterogeneous disease, which requires a combination of multiple genetic defects together with environmental influences for disease to develop (34). To this effect and supported by our analysis of CD11b-deficient mice, loss-of-function SNPs in the *ITGAM* locus, while conferring elevated susceptibility to development of autoimmune disease, likely work in synergy with other predisposing mutations to drive disease.

Lyn is a major regulator of B cell responses (35), with B cells central to the development of inflammation and autoimmune pathology in clinical and experimental lupus (2, 36, 37). A key hallmark of immune dysregulation in *Lyn^-/-^* mice is severe B lymphopenia, a trait that is intrinsically linked to Lyn-deficiency, accompanied by expansion of plasma cells (20, 22, 32). These defects persist in *Lyn^-/-^Itgam^-/-^* mice, yet autoantibody titers were augmented, hinting that CD11b-deficiency may further promote autoimmunity. Interestingly, a mechanism by which CD11b can intrinsically restrain autoreactivity in B cells involves the Lyn-CD22-SHP-1 regulatory axis (14); therefore it is unsurprising that loss of two regulatory mediators in this model, Lyn and CD11b, exacerbated autoantibody responses. This also coincided with further enhanced skewing to effector CD4^+^ T cells, which are required for autoantibody production and disease manifestation in Lyn^-/-^ mice (31, 38). While loss of CD11b may intrinsically promote T cell activation (39), it is likely the enhanced hyper-maturation observed in *Lyn^-/-^Itgam^-/-^* T cells is driven by further dysregulation of inflammatory signaling due to loss of CD11b-mediated regulation of the myeloid compartment (15–17, 31). Engagement of CD11b can induce inhibitory signaling pathways to suppress myeloid cell activation, and importantly these regulatory pathways are facilitated by Src family kinases, namely Lyn (15–17). We observed that the combined loss of Lyn and CD11b accelerated myeloid dysregulation systemically, and in the diseased kidney, manifesting as increased expansion of inflammatory macrophages and enhanced local inflammatory cytokine responses. This suggests that loss of CD11b reduces the threshold for activating inflammatory signals and reinforces an immunoregulatory role for CD11b in the myeloid compartment in lupus-like disease. In line with this, monocytes harboring the R77H loss-of-function CD11b variant also lost the ability to regulate cytokine production *via* iC3b engagement (19), further supporting a propensity for aberrant inflammatory signaling in the absence of functional CD11b.

Strikingly, in conjunction with increased populations of effector immune cells in the chronically inflamed kidney, we observed unperturbed leukocyte recruitment and interestingly, greater glomerular neutrophil adhesion in *Lyn^-/-^Itgam^-/-^* mice. This is notable as we and others have previously established that CD11b is important for effective recruitment of neutrophils and the induction of their responses in acute glomerular inflammation (28, 40). In this context, CD11a is potentially underpinning inflammatory leukocyte trafficking, as we see that it is highly expressed on neutrophils and markedly upregulated on both peripheral monocytes and local kidney macrophages, driven by loss of Lyn and independent of CD11b expression. This notion is supported by previous work in a passive lupus serum transfer nephritis model, which is FcγR-dependent and immune complex-mediated, showing that loss of CD11b facilitates glomerulonephritis *via* enhanced neutrophil vascular rolling and adhesion (41), indicating that CD11b may in fact regulate leukocyte trafficking under these conditions. Furthermore, these trafficking processes were completely lost with CD18-deficiency, attributed to the lack of functional LFA-1 (CD11a/CD18) (41), suggesting that LFA-1 may be sufficient to mediate trafficking during inflammation. The Mac-1 complex has been described to directly interact with the low-affinity, activating FcγRIIa (FcγRIII in mice), inhibiting their binding with immune complex, limiting glomerular neutrophil influx in an autoantibody induced nephritis model (42). Of note, the lupus-associated R77H *ITGAM* variant lacks this capacity to inhibit FcγR (42), suggesting regulation of immune complex-mediated leukocyte trafficking could be impaired in individuals with these mutations. Interestingly, in *in vitro* experimental systems, SLE-associated CD11b variants impaired non-immunoglobulin ligand-induced monocyte static adherence and neutrophil adherence under flow (18, 19). This highlights the contextual importance of the *in vivo* inflammatory environment in supporting leukocyte recruitment processes. Cell surface expression of CD11a was not reported with these variants, so it is unclear whether the upregulation we observed in our model is similarly observed on human monocytes with disease, or potentially, whether the *in vitro* processing and assessment of cells influenced their integrin expression or function. Furthermore, the interaction between loss-of-function CD11b variants and vascular adhesion molecules may block functional LFA-1 from binding, thus inhibiting leukocyte-endothelial attachment, which would not occur in a system such as ours, where CD11b is absent. Nonetheless, the fact that CD11b was dispensable for leukocyte recruitment to the kidney in our *in vivo* model reinforces the capacity for redundancy amongst integrins, but also that concomitant mutations and the disease-specific inflammatory environment may influence the ability of leukocyte trafficking molecules to drive tissue migration.

A previous study examined the role of leukocyte integrins on pathology and survival in the naturally occurring lupus-prone mouse model, MRL/MpJ-Fas^lpr^, which harbors a loss-of-function mutation in the cell-death receptor *Fas*. Contrary to the exacerbation of kidney pathology and impaired survival we observed in *Lyn-/-Itgam-/-* mice, deficiency of CD11b in MRL/MpJ-Fas^lpr^ mice conferred no pathological or clinical differences (43). This could be due to more rapid development of disease and rate of mortality of MRL/MpJ-Fas^lpr^ mice and the primary focus of the study on end-stage disease, rather than the progressive stages of developing disease we assessed in this study. Alternatively, the specific combination of deleting *Itgam* concurrently with *Lyn*, which as discussed prior, are both key regulators of B cell autoreactivity and myeloid cell activation, may have a greater influence on disease than concurrent lymphoproliferative *Fas* mutations. This highlights the importance of interactions between specific disease susceptibility genes as being more influential to disease than the mere accumulation of genetic mutations in lupus.

Interestingly, a recent study has shown that mice expressing constitutively active CD11b exhibited reduced myeloid cell recruitment during peritonitis and were protected against atherosclerosis by limiting macrophage infiltration in lesions (44), suggesting agonism of CD11b may restrict leukocyte trafficking to sites of inflammation and protect from immunopathology. This has been recapitulated therapeutically in pre-clinical studies with the CD11b agonist leukadherin-1, which prevented kidney allograft rejection due to impaired leukocyte infiltration (45) and protected against glomerulonephritis in an induced injury model (46). Leukadherin-1 has also been shown to restrict the activation of the NF-κB and proinflammatory cytokine synthesis pathways, rescuing the MRL/MpJ-Fas^lpr^ lupus model from skin and renal pathology (47), supporting further exploration of CD11b agonism as a therapeutic approach for lupus and other nephritic diseases.

In summary, we have determined that CD11b-deficiency acts pathogenically in lupus to promote inflammatory responses and autoimmune disease on a susceptible genetic background, accelerating immune cell defects and autoimmune kidney pathology, and we have shown that leukocyte trafficking to the chronically inflamed glomerulus can occur in a CD11b-independent manner. This highlights a primarily immunoregulatory role for CD11b in lupus and supports further assessment of the CD11b pathway as a potential therapeutic avenue for lupus nephritis.

## Data Availability Statement

The raw data supporting the conclusions of this article will be made available by the authors, without undue reservation.

## Ethics Statement

The animal studies were reviewed and approved by the Alfred Research Alliance and Monash Medical Centre B Animal Ethics Committees.

## Author Contributions

MLH conceived the study; TG, MJH and MLH designed research; TG, PH, ET and EL-S performed research; TG and PH analyzed data; MJH contributed experimental tools; MLH and MJH provided funding; TG wrote the manuscript; MLH and MJH provided editorial input and all other authors provided editorial comment. All authors contributed to the article and approved the submitted version.

## Funding

This work was supported by funding from the National Health and Medical Research Council of Australia [1080274 to MLH, 1124459 to MJH] and the Central Clinical School, Monash University. TG was a recipient of an Australian Postgraduate Award for doctoral studies, and MJH held a fellowship from the National Health and Medical Research Council Australia [1135971].

## Conflict of Interest

The authors declare that the research was conducted in the absence of any commercial or financial relationships that could be construed as a potential conflict of interest.

## Publisher’s Note

All claims expressed in this article are solely those of the authors and do not necessarily represent those of their affiliated organizations, or those of the publisher, the editors and the reviewers. Any product that may be evaluated in this article, or claim that may be made by its manufacturer, is not guaranteed or endorsed by the publisher.

## Supplementary Material

Supplementary Figure 1 CD11b expression is dysregulated in *Lyn^-/-^* mice and autoimmune pathology manifests in *Lyn^-/-^Itgam^-/-^* mice. **(A)** Flow cytometry assessment of CD11b expression on splenic neutrophils (CD11b^+^Ly6G^+^) of the indicated mice at 12-, 24- and 36-weeks of age; **(B)** Representative histopathology depicting glomeruli of the indicated 36-week-old mice with quantitation of glomerular cross-sectional area. For **(A)**, histograms are representative, and data are derived from n = 7-12 per group, pooled from 3-4 experiments per time-point. The CD11b-ve histogram was derived by gating on the population of C57BL/6 cells that do not express CD11b. For **(B)**, images are representative of four experiments and data are of n = 8-11 per group. **p < 0.01, ***p < 0.001 by Dunn’s multiple comparisons test and ^####^p < 0.0001 by Mann-Whitney U test.

Supplementary Figure 2 The B cell compartment in *Lyn^-/-^Itgam^-/-^* mice is unchanged from that in *Lyn^-/-^* mice. Spleens from 24-week-old mice of the indicated genotypes were assessed by flow cytometry for: **(A)** populations of B cells (B220+CD138-) and plasma cells (CD138+B220-) cells; **(B)** numbers of B cells with **(C)** proportions of follicular (Fol.; CD23^+^CD21^low^), marginal zone (MZ; CD21^+^CD23^-^) and transitional stage 1-3 (T1-3; CD23^-^CD21^-^) subsets; **(D)** numbers of plasma cells, and relative surface expression of **(E)** IgM and **(F)** FcγRII on plasma cells. Flow plots in **(A)** are representative of three experiments. For data in **(B-F)**, n = 7-12 per group, pooled from three experiments. *p < 0.05, **p < 0.01, ***p < 0.001, ****p < 0.0001 by Dunn’s multiple comparisons test and ^#^ p < 0.05, ^##^ p < 0.01 by Mann-Whitney U test.

Supplementary Figure 3 Kidney flow cytometry gating strategy. Perfused and digested kidneys from 24-week-old mice of the indicated genotypes were assessed by flow cytometry for: **(A)** endothelial cells (CD45^-^CD31^+^EpCAM^-^); leukocytes (CD45^+^); lymphocyte populations (CD45^+^CD11b^-^CD11c^-^) including: γδ T cells (CD3ϵ^+^γδTCR^+^), CD4+ T cells (γδTCR^-^CD3ϵ^+^CD4^+^), CD8+ T cells (γδTCR^-^CD4^-^CD3ϵ^+^CD8α^+^) and B cells (γδTCR^-^CD3ϵ^-^CD4^-^CD8α^-^CD19^+^); granulocyte populations (CD45^+^CX3CR1^-^F4/80^-^) including: neutrophils (Ly6G^+^SiglecF^+^) and eosinophils (SiglecF^+^Ly6G^-^); and phagocyte populations (CD45^+^CX3CR1^+^F4/80^+^), further subdivided in . Flow cytometric assessment of the expression of **(B)** adhesion molecules VCAM-1 and ICAM-1 on kidney endothelium and **(C)** integrins CD11a (LFA-1) and CD11c on kidney neutrophils. Flow plots in **(A)** are illustrative of gating strategy only, flow plots in **(B, C)** are representative of two experiments. Data in **(A)** are n = 5-9 per group and data in **(B)** are n = 6-9 per group, both pooled from two experiments. *p < 0.05, **p < 0.01 by Dunn’s multiple comparisons test and p value stated if approaching significance (p < 0.1), ^##^p < 0.01, by Mann-Whitney U test

Supplementary Figure 4 Immune complex deposition is not further enhanced by CD11b-deficiency in Lyn-/- mice. Immunofluorescence analysis and quantification of frozen kidney sections stained for glomerular IgG deposition. Images are representative and data is of n = 6-9 per group, scored blinded. **p < 0.01 by Dunn’s multiple comparisons test.

## References

[1] Vincent FB Figgett WA Hibbs ML. Hallmark of Systemic Lupus Erythematosus: Role of B Cell Hyperactivity. In: Hoi A, editor. Pathogenesis of Systemic Lupus Erythematosus: Insights From Translational Research. Cham: Springer International Publishing (2021). p. 9–36.

[2] Gottschalk TA Tsantikos E Hibbs ML. Pathogenic Inflammation and Its Therapeutic Targeting in Systemic Lupus Erythematosus. Front Immunol (2015) 6(550). doi: 10.3389/fimmu.2015.00550 PMC4623412 26579125

[3] Parikh SV Almaani S Brodsky S Rovin BH. Update on Lupus Nephritis: Core Curriculum 2020. Am J Kidney Dis (2020) 76(2):265–81. doi: 10.1053/j.ajkd.2019.10.017 32220510

[4] Hom G Graham RR Modrek B Taylor KE Ortmann W Garnier S. Association of Systemic Lupus Erythematosus With C8orf13-BLK and ITGAM-ITGAX. N Engl J Med (2008) 358(9):900–9. doi: 10.1056/NEJMoa0707865 18204098

[5] Nath SK Han S Kim-Howard X Kelly JA Viswanathan P Gilkeson GS. A Nonsynonymous Functional Variant in Integrin-Alpha(M) (Encoded by ITGAM) is Associated With Systemic Lupus Erythematosus. Nat Genet (2008) 40(2):152–4. doi: 10.1038/ng.71 18204448

[6] Harley JB Alarcon-Riquelme ME Criswell LA Jacob CO Kimberly RP Moser KL. Genome-Wide Association Scan in Women With Systemic Lupus Erythematosus Identifies Susceptibility Variants in ITGAM, PXK, KIAA1542 and Other Loci. Nat Genet (2008) 40(2):204–10. doi: 10.1038/ng.81 PMC3712260 18204446

[7] Ross GD. Role of the Lectin Domain of Mac-1/CR3 (CD11b/CD18) in Regulating Intercellular Adhesion. Immunol Res (2002) 25(3):219–27. doi: 10.1385/IR:25:3:219 12018461

[8] Li Z. The alphaMbeta2 Integrin and its Role in Neutrophil Function. Cell Res (1999) 9(3):171–8. doi: 10.1038/sj.cr.7290015 10520599

[9] Lu H Smith CW Hughs BJ Bullard DC Perrard JL Ballantyne C. LFA-1 is Sufficient in Mediating Neutrophil Transmigration in Mac-1 Knockout Mice. FASEB J (1996) 10(6):1621–. doi: 10.1172/JCI119293

[10] Lu HF Smith CW Perrard J Bullard D Tang LP Shappell SB. LFA-1 is Sufficient in Mediating Neutrophil Emigration in Mac-1-Deficient Mice. J Clin Invest (1997) 99(6):1340–50. doi: 10.1172/JCI119293 PMC507950 9077544

[11] Phillipson M Heit B Colarusso P Liu L Ballantyne CM Kubes P. Intraluminal Crawling of Neutrophils to Emigration Sites: A Molecularly Distinct Process From Adhesion in the Recruitment Cascade. J Exp Med (2006) 203(12):2569–75. doi: 10.1084/jem.20060925 PMC2118150 17116736

[12] Ehlers MRW. CR3: A General Purpose Adhesion-Recognition Receptor Essential for Innate Immunity. Microbes Infect (2000) 2(3):289–94. doi: 10.1016/S1286-4579(00)00299-9 10758405

[13] Rosetti F Mayadas TN. The Many Faces of Mac-1 in Autoimmune Disease. Immunol Rev (2016) 269(1):175–93. doi: 10.1111/imr.12373 26683153

[14] Ding C Ma Y Chen X Liu M Cai Y Hu X. Integrin CD11b Negatively Regulates BCR Signalling to Maintain Autoreactive B Cell Tolerance. Nat Commun (2013) 4:2831. doi: 10.1038/ncomms3813 24264377

[15] Wang L Gordon RA Huynh L Su X Park Min KH Han J. Indirect Inhibition of Toll-Like Receptor and Type I Interferon Responses by ITAM-Coupled Receptors and Integrins. Immunity (2010) 32(4):518–30. doi: 10.1016/j.immuni.2010.03.014 PMC2862476 20362473

[16] Han C Jin J Xu S Liu H Li N Cao X. Integrin CD11b Negatively Regulates TLR-Triggered Inflammatory Responses by Activating Syk and Promoting Degradation of MyD88 and TRIF *via* Cbl-B. Nat Immunol (2010) 11(8):734–42. doi: 10.1038/ni.1908 20639876

[17] Zhang Q Lee WB Kang JS Kim LK Kim YJ. Integrin CD11b Negatively Regulates Mincle-Induced Signaling *via* the Lyn-SIRPα-SHP1 Complex. Exp Mol Med (2018) 50(2):e439. doi: 10.1038/emm.2017.256 29400702 PMC5992981

[18] Zhou Y Wu J Kucik DF White NB Redden DT Szalai AJ. Multiple Lupus-Associated ITGAM Variants Alter Mac-1 Functions on Neutrophils. Arthritis Rheum (2013) 65(11):2907–16. doi: 10.1002/art.38117 PMC3969028 23918739

[19] Rhodes B Furnrohr BG Roberts AL Tzircotis G Schett G Spector TD. The Rs1143679 (R77H) Lupus Associated Variant of ITGAM (CD11b) Impairs Complement Receptor 3 Mediated Functions in Human Monocytes. Ann Rheum Dis (2012) 71(12):2028–34. doi: 10.1136/annrheumdis-2012-201390 PMC3488763 22586164

[20] Hibbs ML Tarlinton DM Armes J Grail D Hodgson G Maglitto R. Multiple Defects in the Immune System of Lyn-Deficient Mice, Culminating in Autoimmune Disease. Cell (1995) 83(2):301–11. doi: 10.1016/0092-8674(95)90171-X 7585947

[21] Tsantikos E Gottschalk TA Maxwell MJ Hibbs ML. Role of the Lyn Tyrosine Kinase in the Development of Autoimmune Disease. Int J Clin Rheumatol (2014) 9(5):519–35. doi: 10.2217/ijr.14.44

[22] Gottschalk TA Vincent FB Hoi AY Hibbs ML. Granulocyte Colony-Stimulating Factor is Not Pathogenic in Lupus Nephritis. Immun Inflamm Dis (2021) 9(3):758–70. doi: 10.1002/iid3.430 PMC8342225 33960699

[23] Coxon A Rieu P Barkalow FJ Askari S Sharpe AH vonAndrian UH. A Novel Role for the Beta 2 Integrin CD11b/CD18 in Neutrophil Apoptosis: A Homeostatic Mechanism in Inflammation. Immunity (1996) 5(6):653–66. doi: 10.1016/S1074-7613(00)80278-2 8986723

[24] Ruiz-Villalba A Mattiotti A Gunst QD Cano-Ballesteros S van den Hoff MJ Ruijter JM. Reference Genes for Gene Expression Studies in the Mouse Heart. Sci Rep (2017) 7(1):24. doi: 10.1038/s41598-017-00043-9 28154421 PMC5428317

[25] Overbergh L Giulietti A Valckx D Decallonne R Bouillon R Mathieu C. The Use of Real-Time Reverse Transcriptase PCR for the Quantification of Cytokine Gene Expression. J Biomol Tech (2003) 14(1):33–43. 12901609 PMC2279895

[26] Qian BZ Li J Zhang H Kitamura T Zhang J Campion LR. CCL2 Recruits Inflammatory Monocytes to Facilitate Breast-Tumour Metastasis. Nature (2011) 475(7355):222–5. doi: 10.1038/nature10138 PMC3208506 21654748

[27] De Filippo K Henderson RB Laschinger M Hogg N. Neutrophil Chemokines KC and Macrophage-Inflammatory Protein-2 are Newly Synthesized by Tissue Macrophages Using Distinct TLR Signaling Pathways. J Immunol (2008) 180(6):4308–15. doi: 10.4049/jimmunol.180.6.4308 18322244

[28] Devi S Li A Westhorpe CL Lo CY Abeynaike LD Snelgrove SL. Multiphoton Imaging Reveals a New Leukocyte Recruitment Paradigm in the Glomerulus. Nat Med (2013) 19(1):107–12. doi: 10.1038/nm.3024 23242472

[29] Finsterbusch M Hall P Li A Devi S Westhorpe CL Kitching AR. Patrolling Monocytes Promote Intravascular Neutrophil Activation and Glomerular Injury in the Acutely Inflamed Glomerulus. Proc Natl Acad Sci U S A (2016) 113(35):E5172–81. doi: 10.1073/pnas.1606253113 PMC5024581 27528685

[30] Yeung L Gottschalk TA Hall P Tsantikos E Gallagher RH Kitching AR. Tetraspanin CD53 Modulates Lymphocyte Trafficking But Not Systemic Autoimmunity in Lyn-Deficient Mice. Immunol Cell Biol (2021) 99(10):1053–66. doi: 10.1111/imcb.12501 34514627

[31] Maxwell MJ Tsantikos E Kong AM Vanhaesebroeck B Tarlinton DM Hibbs ML. Attenuation of Phosphoinositide 3-Kinase Delta Signaling Restrains Autoimmune Disease. J Autoimmun (2012) 38(4):381–91. doi: 10.1016/j.jaut.2012.04.001 22537464

[32] Tsantikos E Oracki SA Quilici C Anderson GP Tarlinton DM Hibbs ML. Autoimmune Disease in Lyn-Deficient Mice is Dependent on an Inflammatory Environment Established by IL-6. J Immunol (2010) 184(3):1348–60. doi: 10.4049/jimmunol.0901878 20042579

[33] Lin SL Castaño AP Nowlin BT Lupher ML Jr. Duffield JS. Bone Marrow Ly6Chigh Monocytes are Selectively Recruited to Injured Kidney and Differentiate Into Functionally Distinct Populations. J Immunol (2009) 183(10):6733–43. doi: 10.4049/jimmunol.0901473 19864592

[34] Ghodke-Puranik Y Niewold TB. Immunogenetics of Systemic Lupus Erythematosus: A Comprehensive Review. J Autoimmun (2015) 64:125–36. doi: 10.1016/j.jaut.2015.08.004 PMC4628859 26324017

[35] Xu Y Harder KW Huntington ND Hibbs ML Tarlinton DM. Lyn Tyrosine Kinase: Accentuating the Positive and the Negative. Immunity (2005) 22(1):9–18. doi|: 10.1016/j.immuni.2004.12.004 15664155

[36] Lamagna C Hu Y DeFranco AL Lowell CA. B Cell-Specific Loss of Lyn Kinase Leads to Autoimmunity. J Immunol (2014) 192(3):919–28. doi: 10.4049/jimmunol.1301979 PMC3900234 24376269

[37] Ma K Du W Wang X Yuan S Cai X Liu D. Multiple Functions of B Cells in the Pathogenesis of Systemic Lupus Erythematosus. Int J Mol Sci (2019) 20(23):6021. doi: 10.3390/ijms20236021 PMC6929160 31795353

[38] Hua Z Gross AJ Lamagna C Ramos-Hernandez N Scapini P Ji M. Requirement for MyD88 Signaling in B Cells and Dendritic Cells for Germinal Center Anti-Nuclear Antibody Production in Lyn-Deficient Mice. J Immunol (2014) 192(3):875–85. doi: 10.4049/jimmunol.1300683 PMC4101002 24379120

[39] Wagner C Hansch GM Stegmaier S Denefleh B Hug F Schoels M. The Complement Receptor 3, CR3 (CD11b/CD18), on T Lymphocytes: Activation-Dependent Up-Regulation and Regulatory Function. Eur J Immunol (2001) 31(4):1173–80. doi: 10.1002/1521-4141(200104)31:4<1173::AID-IMMU1173>3.0.CO;2-9 11298342

[40] Tang T Rosenkranz A Assmann KJ Goodman MJ Gutierrez-Ramos JC Carroll MC. A Role for Mac-1 (CDIIb/CD18) in Immune Complex-Stimulated Neutrophil Function *In Vivo*: Mac-1 Deficiency Abrogates Sustained Fcgamma Receptor-Dependent Neutrophil Adhesion and Complement-Dependent Proteinuria in Acute Glomerulonephritis. J Exp Med (1997) 186(11):1853–63. doi: 10.1084/jem.186.11.1853 PMC2211718 9382884

[41] Rosetti F Tsuboi N Chen K Nishi H Ernandez T Sethi S. Human Lupus Serum Induces Neutrophil-Mediated Organ Damage in Mice That Is Enabled by Mac-1 Deficiency. J Immunol (2012) 189(7):3714–23. doi: 10.4049/jimmunol.1201594 PMC3462585 22933624

[42] Saggu G Okubo K Chen Y Vattepu R Tsuboi N Rosetti F. Cis Interaction Between Sialylated Fcγriia and the αi-Domain of Mac-1 Limits Antibody-Mediated Neutrophil Recruitment. Nat Commun (2018) 9(1):5058. doi: 10.1038/s41467-018-07506-1 30498196 PMC6265255

[43] Kevil CG Hicks MJ He X Zhang J Ballantyne CM Raman C. Loss of LFA-1, But Not Mac-1, Protects MRL/MpJ-Fas(lpr) Mice From Autoimmune Disease. Am J Pathol (2004) 165(2):609–16. doi: 10.1016/S0002-9440(10)63325-1 PMC1618580 15277234

[44] Martinez L Li X Ramos-Echazabal G Faridi H Zigmond ZM Santos Falcon N. A Genetic Model of Constitutively Active Integrin CD11b/CD18. J Immunol (2020) 205(9):2545–53. doi: 10.4049/jimmunol.1901402 PMC7577938 32938725

[45] Khan SQ Guo L Cimbaluk DJ Elshabrawy H Faridi MH Jolly M. A Small Molecule β2 Integrin Agonist Improves Chronic Kidney Allograft Survival by Reducing Leukocyte Recruitment and Accompanying Vasculopathy. Front Med (Lausanne) (2014) 1:45. doi: 10.3389/fmed.2014.00045 25593918 PMC4291902

[46] Maiguel D Faridi MH Wei C Kuwano Y Balla KM Hernandez D. Small Molecule-Mediated Activation of the Integrin CD11b/CD18 Reduces Inflammatory Disease. Sci Signal (2011) 4(189):ra57. doi: 10.1126/scisignal.2001811 21900205 PMC4507414

[47] Faridi MH Khan SQ Zhao W Lee HW Altintas MM Zhang K. CD11b Activation Suppresses TLR-Dependent Inflammation and Autoimmunity in Systemic Lupus Erythematosus. J Clin Invest (2017) 127(4):1271–83. doi: 10.1172/JCI88442 PMC5373862 28263189

