# Supplementary material for: Loss of CD11b Accelerates Lupus Nephritis in Lyn-Deficient Mice Without Disrupting Glomerular Leukocyte Trafficking
Source: Front Immunol. 2022 May 12;13:875359. doi: 10.3389/fimmu.2022.875359 (PMC9134083; doi:10.3389/fimmu.2022.875359)
Supplement: Supplementary Figure 1 — CD11b expression is dysregulated in Lyn-/- mice and autoimmune pathology manifests in Lyn-/-Itgam-/- mice. (A) Flow cytometry assessment of CD11b expression on splenic neutrophils (CD11b+Ly6G+) of the indicated mice at 12-, 24- and 36-weeks of age; (B) Representative histopathology depicting glomeruli of the indicated 36-week-old mice with quantitation of glomerular cross-sectional area. For (A), histograms are representative, and data are derived from n = 7-12 per group, pooled from 3-4 experiments per time-point. The CD11b-ve histogram was derived by gating on the population of C57BL/6 cells that do not express CD11b. For (B), images are representative of four experiments and data are of n = 8-11 per group. **p < 0.01, ***p < 0.001 by Dunn’s multiple comparisons test and ####p < 0.0001 by Mann-Whitney U test. [file DataSheet_1.pdf]

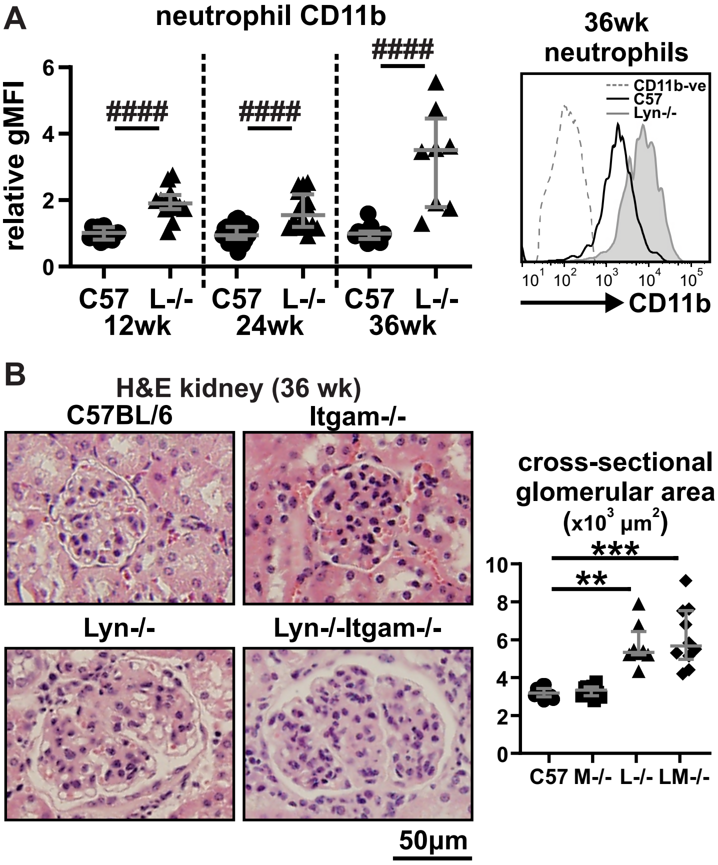

SUPPLEMENTARY FIGURE 1. CD11b expression is dysregulated in *Lyn*$^{-/-}$ mice and autoimmune pathology manifests in *Lyn*$^{-/-}$*Itgam*$^{-/-}$ mice.

(A) Flow cytometry assessment of CD11b expression on neutrophils of the indicated mice at 12-, 24- and 36-weeks of age; **(B)** Representative histopathology depicting glomeruli of the indicated 36-week-old mice with quantitation of glomerular cross-sectional area. For **A**, histograms are representative and data are derived from n=7-12 per group, pooled from 3-4 experiments per time-point. The CD11b-ve histogram was derived by gating on the population of C57BL/6 cells that do not express CD11b. For **B**, images are representative of four experiments and data are of n=8-11 per group. ** p < 0.01, *** p < 0.001 by Dunn's multiple comparisons test and #### p < 0.0001 by Mann-Whitney U test.

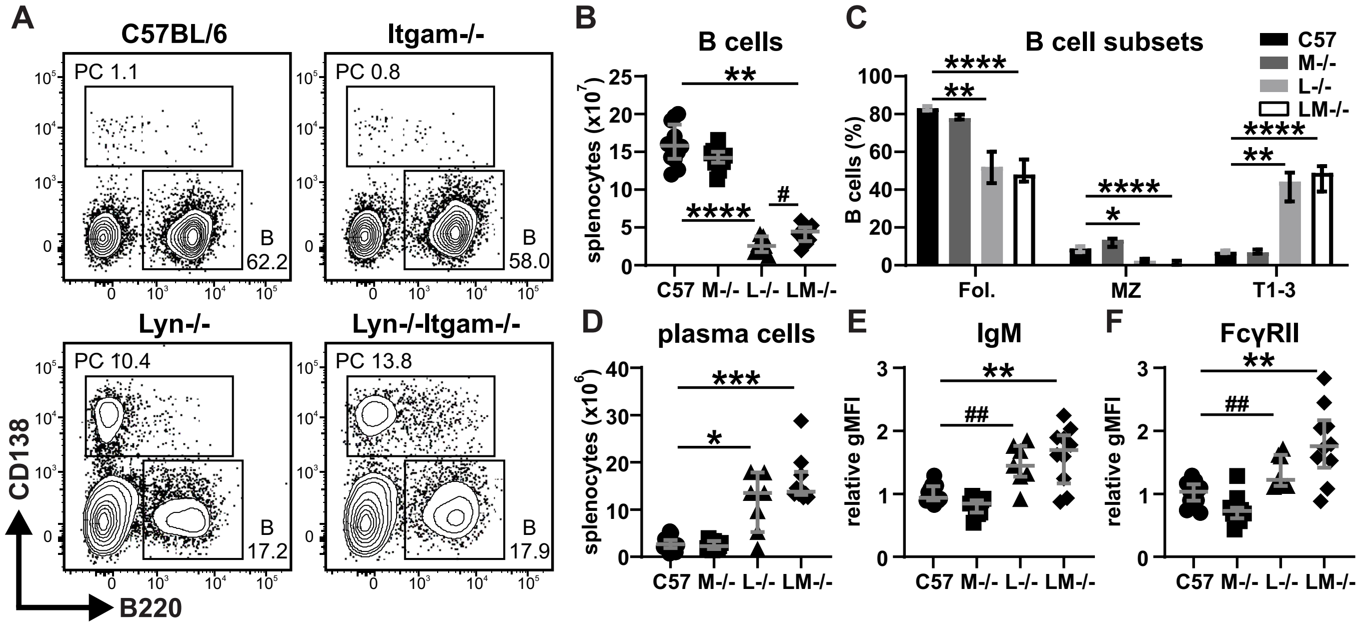

SUPPLEMENTARY FIGURE 2. The B cell compartment is unaffected by loss of CD11b in *Lyn*$^{-/-}$ mice.

Spleens from 24-week-old mice of the indicated genotypes were assessed by flow cytometry for: **(A)** populations of B cells (B220+CD138-) and plasma cells (CD138+B220-) cells; **(B)** numbers of B cells with **(C)** proportions of follicular (Fol.; $CD23^{+}CD21^{low}$), marginal zone (MZ; $CD21^{+}CD23^{-}$) and transitional stage 1-3 (T1-3; $CD23^{-}CD21^{-}$) subsets; **(D)** numbers of plasma cells with surface expression of **(E)** IgM and **(F)** FcγRII. Flow plots in **A** are representative of three experiments. For data in **B-F**, n=7-12 per group, pooled from three experiments. * p < 0.05, ** p < 0.01, *** p < 0.001, **** p < 0.0001 by Dunn's multiple comparisons test and # p < 0.05, ## p < 0.01 by Mann-Whitney U test.

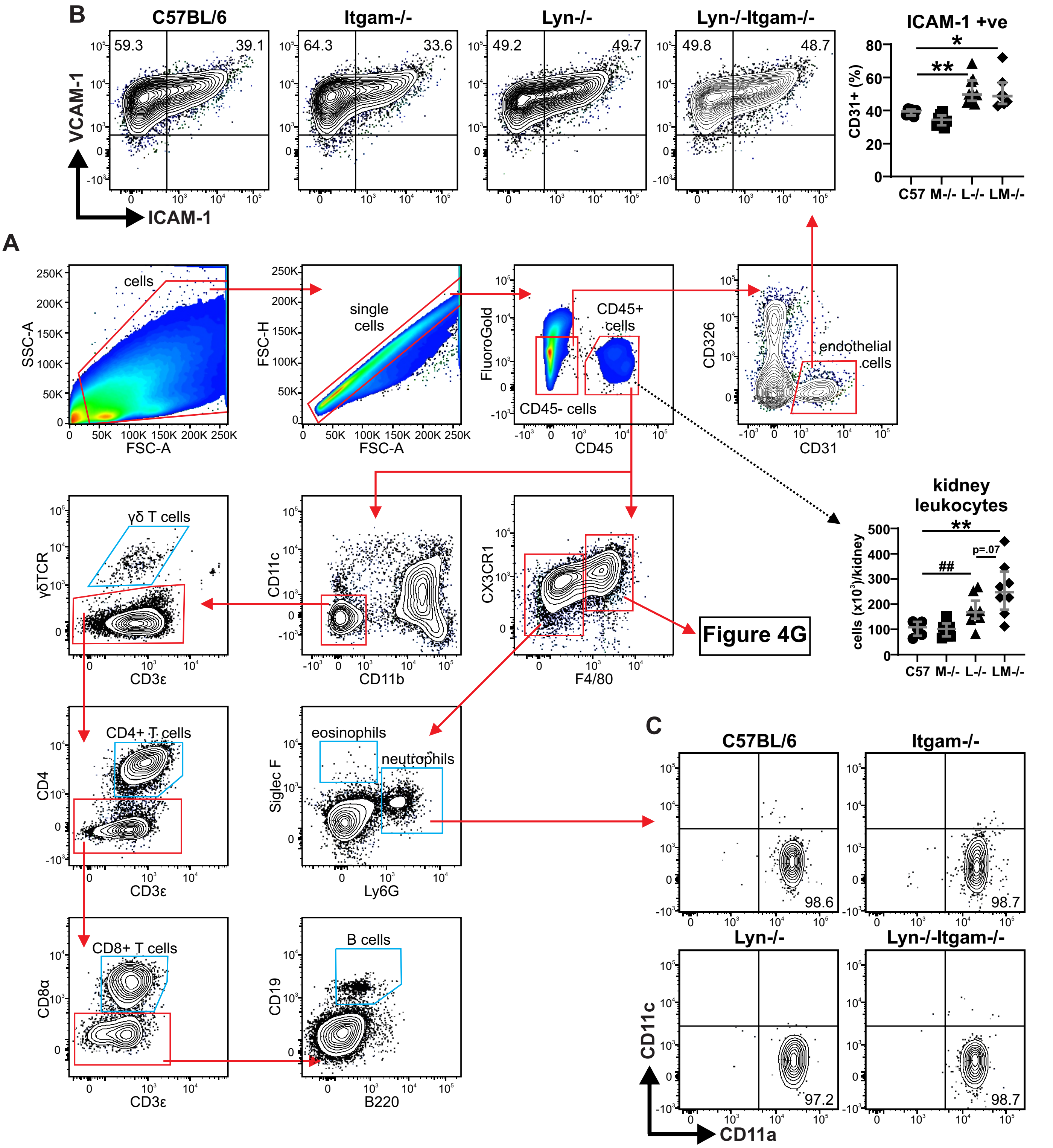

SUPPLEMENTARY FIGURE 3. Kidney flow cytometry gating strategy.

Perfused and digested kidneys from 24-week-old mice of the indicated genotypes were assessed by flow cytometry for: **(A)** endothelial cells ($CD45^-CD31^+EpCAM^-$); leukocytes ($CD45^+$); lymphocyte populations ($CD45^+CD11b^-CD11c^-$) including: γδ T cells ($CD3\varepsilon^+\gamma\delta TCR^+$), CD4+ T cells ($\gamma\delta TCR^-CD3\varepsilon^+CD4^+$), CD8+ T cells ($\gamma\delta TCR^-CD4^-CD3\varepsilon^+CD8\alpha^+$) and B cells ($\gamma\delta TCR^-CD3\varepsilon^-CD4^-CD8\alpha^-CD19^+$); granulocyte populations ($CD45^+CX3CR1^-F4/80^-$) including: neutrophils ($Ly6G^+SiglecF^+$) and eosinophils ($SiglecF^+Ly6G^-$); and phagocyte populations ($CD45^+CX3CR1^+F4/80^+$), further subdivided in Figure 4G. Flow cytometric assessment of the expression of **(B)** adhesion molecules VCAM-1 and ICAM-1 on kidney endothelium and **(C)** integrins CD11a (LFA-1) and CD11c on kidney neutrophils. Flow plots in **A** are illustrative of gating strategy only, flow plots in **B** and **C** are representative of two experiments. Data in **A** are n=5-9 per group and data in **B** are n=6-9 per group, both pooled from two experiments. * $p < 0.05$, ** $p < 0.01$ by Dunn's multiple comparisons test and p value stated if approaching significance ($p < 0.1$), ## $p < 0.01$, by Mann-Whitney U test.

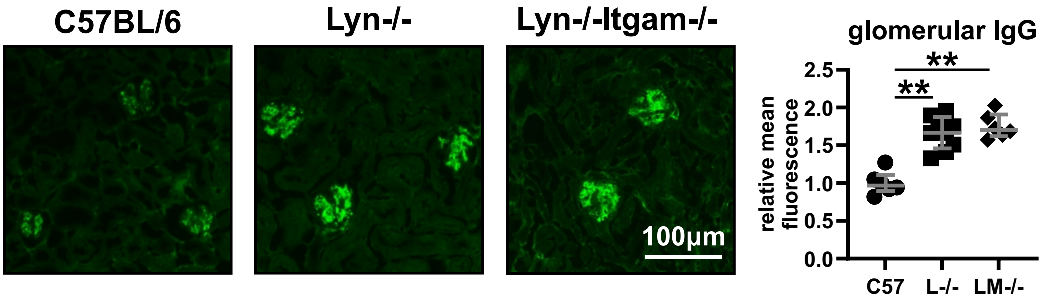

SUPPLEMENTARY FIGURE 4. Immune complex deposition is not further enhanced by CD11b-deficiency in Lyn$^{-/-}$ mice.

Immunofluorescence analysis and quantification of frozen kidney sections for glomerular IgG deposition. Images are representative and data is of n=6-9 per group. ** p < 0.01 by Dunn's multiple comparisons test.
